# Intraoperative OCT observation of incision-related descemet membrane detachment during implantable collamer lens implantation

**DOI:** 10.3389/fmed.2026.1888213

**Published:** 2026-10-15

**Authors:** Jiao Xie, Nian Guan, Ying Liu, Zhenwen Zheng, Xing He, Zhengwei Shen

**Affiliations:** Wuhan Bright Eye Hospital, Wuhan, China

**Keywords:** descement membrane detachment (DMD), implantable collamer lens (ICL), intraoperative optical coherence tomography (OCT) navigation microscope, phakic intraocular lens implantation surgery, retrospective case series

## Abstract

**Purpose:**

The occurrence of Descemet membrane detachment (DMD) during each surgical step of implantable Collamer lens (ICL) surgery for phakic eyes using intraoperative OCT navigation microscope, and to analyze the factors influencing DMD.

**Methods:**

The single-center retrospective case series included 100 eyes of 50 patients aged 21 to 38 years with refractive errors from July to October 2024, and all patients underwent ICL surgery with a 3.0 mm steep-axis surgical incision. During the operation, the intraoperative OCT navigation microscope was used to observe at each step whether the DMD occured at the incision site and whether the range of detachment changed in subsequent steps.

**Results:**

All procedures were completed successfully without intraoperative complications. No DMD was detected by the end of incision creation. The cumulative number of eyes in which DMD had been detected increased to 21 (21%) by the end of ICL insertion, 34 (34%) by the end of ICL positioning, and 45 (45%) by the end of irrigation/aspiration of the viscoelastic agent. In the mixed-effects analysis accounting for the correlation between fellow eyes, lower preoperative intraocular pressure was associated with higher odds of DMD occurrence (odds ratio per 1-mmHg increase, 0.77; 95% CI, 0.61–0.97; *P* = 0.03). None of the evaluated factors was significantly associated with DMD extent.

**Conclusion:**

Intraoperative OCT enabled real-time detection of incision-related DMD developing or extending during ICL surgery. The cumulative occurrence of DMD increased as the procedure progressed, supporting careful instrument manipulation and minimization of repeated contact with the corneal incision throughout surgery.

## Introduction

In recent years, a sreies of refractive surgery such as eye laser sugery, ICL implantation, etc. have become increasingly well-accepted and well-established worldwide. In which, ICL implantation is a good choice for the correction of moderate to high myopia, particularly in patients who are not suitable candidates for corneal refractive procedures (1). By placing a posterior chamber phakic intraocular lens in the ciliary sulcus, ICL surgery offers several advantages over corneal ablation, including preservation of corneal tissue, reversibility, and excellent visual outcomes, with high patient satisfaction reported across multiple long-term follow-up studies (2).

Despite its overall favorable safety profile, ICL implantation is not without complications. Documented adverse events include cataract formation (reported in up to 19% of eyes over extended follow-up), corneal endothelial cell loss ranging from 1.1% to 13.4% within five years, secondary glaucoma due to pupillary block or pigment dispersion, iris atrophy, and, rarely, endophthalmitis (3). Among corneal complications, endothelial cell loss has received considerable attention; however, incision-related corneal injuries during ICL surgery remain relatively undercharacterized.

DMD is a recognized iatrogenic complication of intraocular surgery, most extensively documented in cataract surgery, with reported incidences of approximately 0.5% for phacoemulsification and 2.5% for extracapsular cataract extraction (4, 5). DMD occurs when the DM separates from the overlying posterior corneal stroma, creating a potential space that may fill with aqueous humor or viscoelastic material. While small, peripheral detachments often resolve spontaneously, extensive or centrally located DMD can result in persistent corneal edema, impaired visual recovery, and, if left untreated, permanent corneal decompensation requiring descemetopexy or endothelial keratoplasty (6).

Several intraoperative risk factors for DMD have been identified, including the use of blunt instruments, inappropriate incision geometry (such as oblique, overly anterior, or shelved tunnels), tight incisions that do not accommodate the surgical instrument, forceful or repeated passage of instruments through the wound, and inadvertent injection of balanced salt solution or ophthalmic viscosurgical devices into the stromal–Descemet interface (7). Notably, ICL implantation shares many of these risk factors: the procedure requires a clear corneal incision of approximately 3.0 mm through which a loaded injector cartridge is inserted, followed by controlled delivery of the foldable lens into the anterior chamber and subsequent manipulation to position the haptics in the ciliary sulcus. The repeated passage of instruments and the injector through the corneal tunnel, combined with the injection of viscoelastic agents, creates a plausible mechanical environment for incision-related DMD. Yet, the incidence, morphological characteristics, and clinical course of DMD specifically associated with ICL implantation have not been systematically investigated.

Anterior segment optical coherence tomography (AS-OCT) has become the gold standard for the diagnosis and quantification of DMD, providing high-resolution cross-sectional images that delineate the position, extent, and configuration of the detached membrane (8). However, conventional AS-OCT is typically performed postoperatively, which limits the ability to capture the dynamic onset of DMD during surgery and to correlate its occurrence with specific surgical maneuvers. Intraoperative OCT (iOCT) addresses this limitation by providing real-time, high-resolution imaging of anterior segment structures throughout the surgical procedure. In recent years, iOCT has been increasingly applied in various anterior segment surgeries, including cataract surgery, corneal transplantation, and ICL implantation, where it has demonstrated value in assessing incision architecture, monitoring ICL vaulting intraoperatively, and guiding surgical decision-making (9–11). In the context of DMD, iOCT has shown particular promise in enabling early detection and guiding real-time repositioning maneuvers, as demonstrated in cases of failed descemetopexy where real-time iOCT significantly improved the success rate of DM reattachment.

Despite these advances, the use of iOCT to systematically observe incision-related DMD during ICL implantation remains unexplored. Understanding the intraoperative onset, morphological patterns, and associated surgical factors of DMD in this setting is essential for developing preventive strategies and optimizing surgical technique. Therefore, the present study employed iOCT to continuously observe the corneal incision throughout ICL implantation, aiming to characterize the incidence, morphological features, and intraoperative evolution of incision-related DMD, and to identify potential risk factors associated with its occurrence.

## Materials and methods

### Patients and study design

This single-center, retrospective clinical study included 50 patients (100 eyes) with refractive errors who underwent ICL implantation at Wuhan Bright Eye Hospital (Hubei, China) between July and October 2024. All surgeries were performed through a 3.0-mm steep-axis main corneal incision. Patients were followed up at 1 week and 1 month postoperatively, and all underwent comprehensive preoperative examinations prior to surgery. This study was approved by the Ethics Committee of Wuhan Bright Eye Hospital and conducted in accordance with the principles of the Declaration of Helsinki.

The inclusion criteria were as follows: (1) age between 21 and 45 years; (2) spherical refraction ranging from −1.5 to −18.0 D and cylindrical refraction from −0.25 to −2.0 D; (3) stable refraction for at least 1 year, defined as a change in spherical equivalent (SE) within ±0.5 D over 12 months; (4) corneal endothelial cell density ≥ 2,000 cells/mm^2^ with stable cell morphology (12); (5) anterior chamber depth ≥ 2.80 mm with an open anterior chamber angle; and (6) strong patient motivation for spectacle independence (13, 14). The exclusion criteria were as follows: (1) a history of ocular trauma or disease; (2) systemic medical conditions that could affect visual outcomes; (3) pregnancy or lactation.

### The implantable collamer lens (ICL)

ICL (STAAR Surgical AG, Nidau, Switzerland) is a posterior chamber phakic intraocular lens fabricated from Collamer, a proprietary biocompatible copolymer consisting of purified porcine collagen covalently bonded with hydroxyethyl methacrylate (HEMA). The incorporation of collagen-comprising approximately 0.1% of the material by dry weight-confers exceptional biocompatibility, as the collagen component is recognized as “self” by the ocular immune system, minimizing foreign-body reactions, inflammatory responses, and pigment dispersion that have historically limited earlier generations of phakic lenses. Unlike corneal refractive procedures that permanently ablate corneal stromal tissue, the ICL is implanted in the ciliary sulcus, anterior to the natural crystalline lens, preserving the integrity of the cornea and the eye's accommodative apparatus. This anatomical placement, combined with the material's biocompatibility and optical performance, makes ICL implantation particularly advantageous for patients with moderate to high myopia, thin corneas, or ocular surface disease who are poor candidates for laser refractive surgery (15–20).

### Ocular assessments

All selected patients underwent routine preoperative examinations in accordance with the standard ICL assessment protocol (21, 22). Uncorrected and corrected distance and near visual acuity were recorded, and best-corrected visual acuity was determined by automated refraction and confirmed by manifest refraction. Additional assessments included intraocular pressure (IOP) measurement, tear break-up time, B-scan ocular ultrasonography, corneal endothelial cell density (ECD), macular and optic nerve optical coherence tomography (OCT), IOLMaster biometry, ultrasound biomicroscopy (UBM), Pentacam imaging, and photopic and scotopic pupil measurements. All patients were confirmed to meet the criteria for posterior chamber phakic intraocular lens implantation.

The ICL power and model were selected based on biometric parameters, including anterior chamber depth (ACD) and horizontal white-to-white (HWTW) distance measured with Pentacam, as well as horizontal and vertical sulcus-to-sulcus (STS) distances obtained by UBM. Posterior chamber angle configuration and ciliary body morphology were also considered (23). The appropriate ICL specifications were calculated using the online calculator provided by STAAR Surgical.

Postoperatively, the recorded videos were reviewed to identify and capture images of DMD, and DMD length was measured using ImageJ software. All measurements were performed by a single ophthalmologist.

### Surgical methods

All surgical procedures were performed by a single experienced surgeon. The surgical procedure comprised the following steps: (1) Incision creation: a disposable 3.0 mm keratome was used to create a corneal tunnel incision at the surgical limbus along the marked steep axis. (2) ICL implantation: the ICL was introduced into the anterior chamber using an injector, and an appropriate volume of 15 mg/mL medical-grade sodium hyaluronate gel (Hangzhou Shangjie Technology Co., Ltd., China) was injected to maintain the anterior chamber depth. (3) ICL adjustment: the four haptics of the EVO-ICL were positioned into the ciliary sulcus behind the iris using an adjustment hook. (4) Viscoelastic agent removal: the viscoelastic agent was removed and replaced with a compound sodium chloride solution (Anhui Double-Crane Pharmaceutical Co., Ltd., China) injected into the anterior chamber using an irrigation needle.

All steps were performed under real-time guidance of the RESCAN 700 intraoperative OCT navigation microscope (Carl Zeiss AG, Oberkochen, Germany) in spectral-domain OCT cross-scan mode (scanning depth and length, 2.0 mm). Intraoperative scanning and video recording were performed using the same system. The presence and extent of Descemet membrane detachment (DMD) at the incision site, specifically involving the anterior and posterior lips, were observed and recorded during each of the four surgical steps. Postoperatively, all patients received two drops of tobramycin/dexamethasone (Alcon-Couvreur SA, Puurs, Belgium) instilled into the operated eye for infection prophylaxis.

### Intraoperative measurement of DMD

Intraoperative imaging was performed using the RESCAN 700 system (Carl Zeiss AG, Oberkochen, Germany). During each procedure, surgical videos were recorded while the OCT crosshair was swept systematically from one end of the incision to the other. Postoperatively, the recorded videos were reviewed; when DMD was identified, the corresponding images were captured, and the image depicting the longest DMD observed during surgery was saved for analysis (Figure 1). The location of DMD at the surgical incision was categorised as involving either the anterior lip (Figure 2) or the posterior lip (Figure 3).

**Figure 1:**
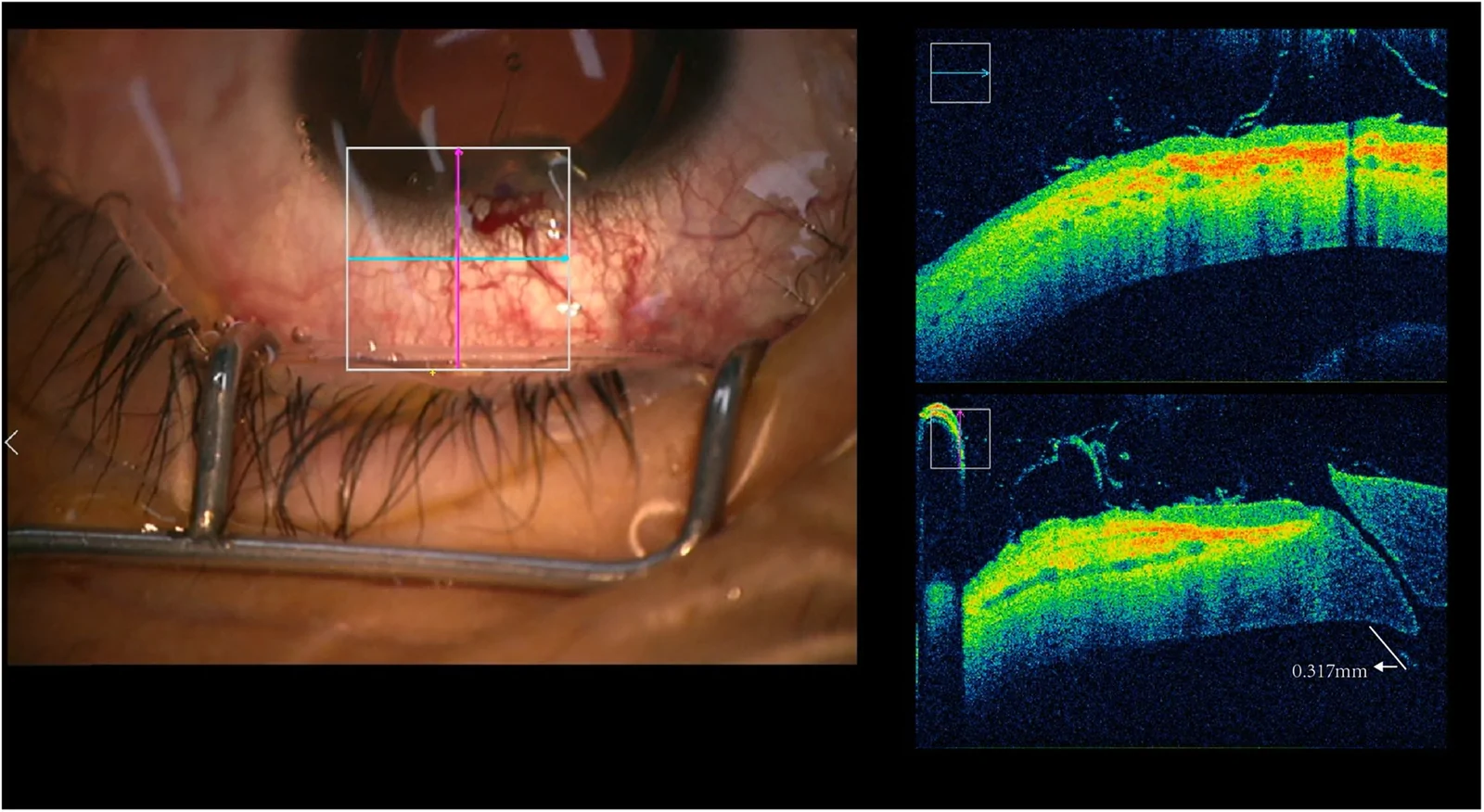
The longest DMD being observed during surgery.

**Figure 2:**
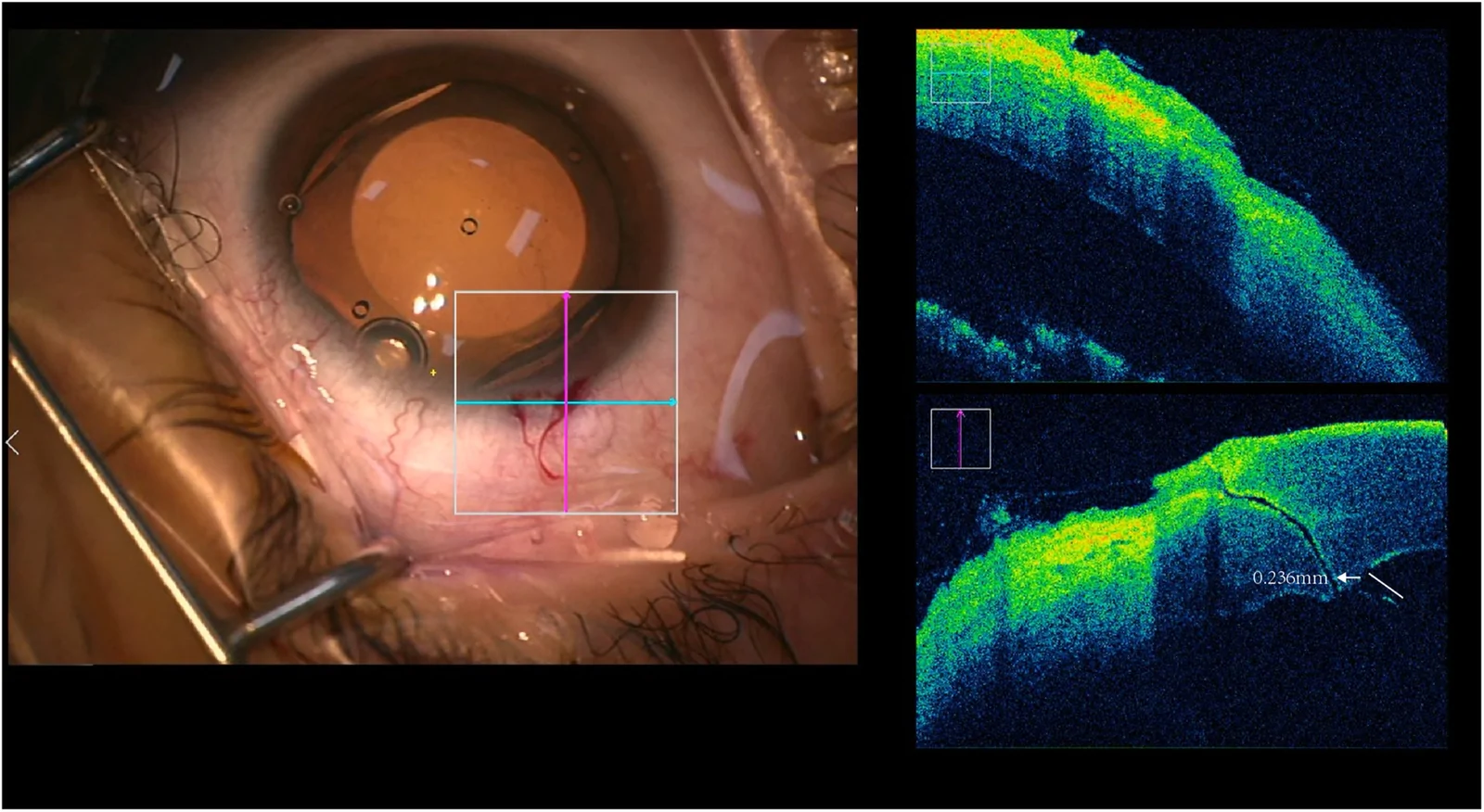
The DMD located at anterior lip.

**Figure 3:**
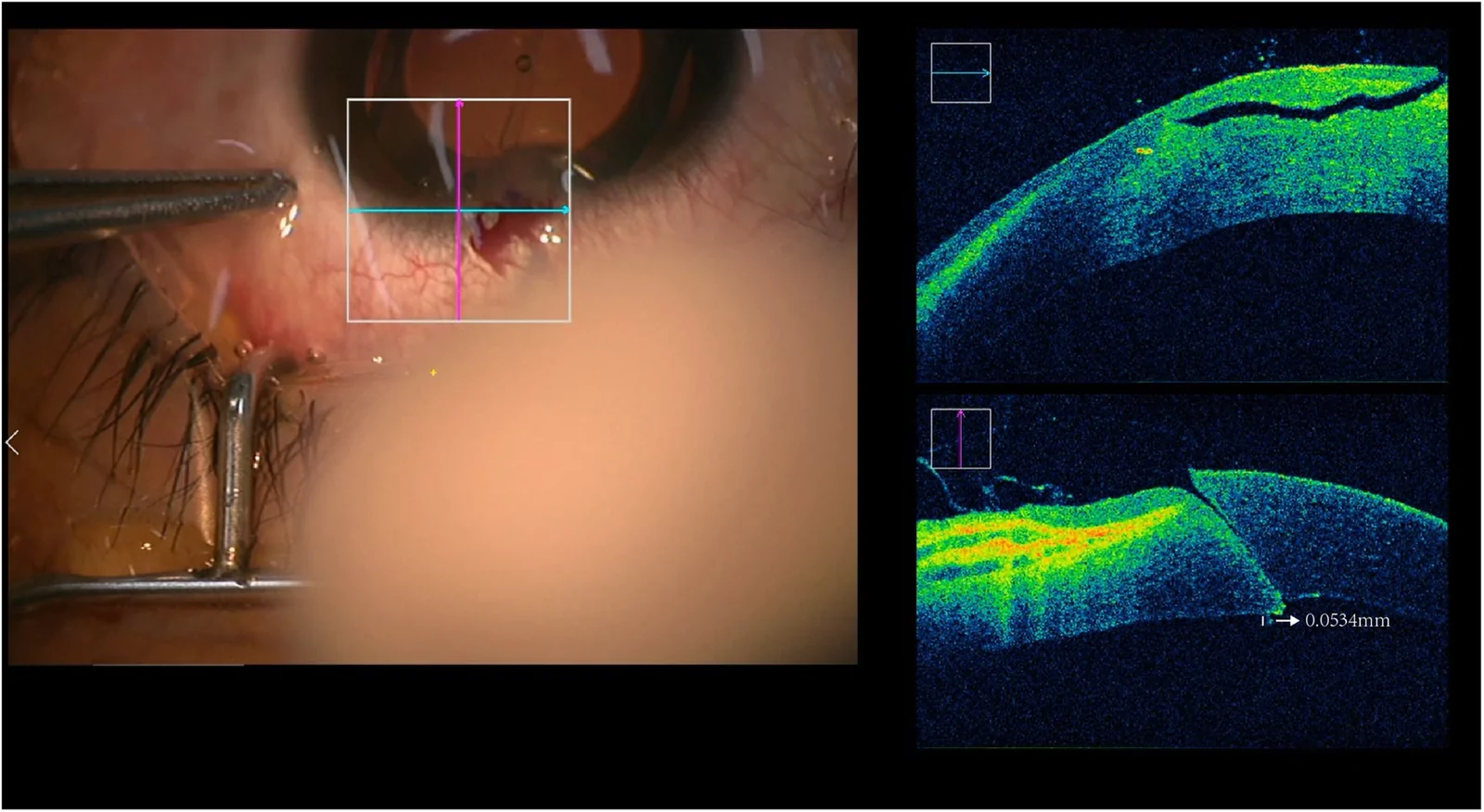
The DMD located at posterior lip.

### Intraoperative DMD range measurement and comparison

Intraoperative DMD images and videos acquired with the intraoperative OCT system were collected and analyzed using ImageJ software (version 1.48). All images exported from the surgical videos were resized to a uniform resolution of 2,469 × 1,349 pixels. All measurements and comparisons were performed by a single observer using the ruler function within ImageJ software (24). This measurement method was used for the qualitative assessment of DMD namely, to determine whether DMD occurred and whether subsequent surgical steps resulted in its extension rather than for quantitative analysis.

### Statistical analysis

Descriptive statistics were used to summarise baseline characteristics and surgical outcomes. Categorical variables were expressed as counts and percentages [n (%)]. Continuous variables that were not normally distributed were summarised as medians and ranges [M (min, max)]. The Wilcoxon signed-rank test was used to evaluate changes in parameters before and after surgery, and the median change with its 95% confidence interval (CI) was calculated to estimate the effect size.

DMD was assessed sequentially during the four surgical stages. Because DMD detected at one stage could persist or extend during subsequent stages, the stage-related results were summarised as the cumulative number and percentage of eyes in which DMD had been detected by the end of each stage; thus, the cumulative value at each later stage included eyes in which DMD had been detected at any preceding stage. This cumulative presentation was adopted to describe the progressive proportion of eyes affected by DMD throughout the surgical workflow, as reporting only newly detected cases at each stage would fail to capture eyes in which previously detected DMD persisted or progressed during later stages. No formal statistical comparisons were performed among the cumulative stage-specific proportions, because these outcomes were nested repeated observations rather than independent stage-specific incidences. Accordingly, the stage-related findings were interpreted descriptively and were not used to estimate the independent effect of any individual surgical stage.

To analyse predictors of the primary outcome—the maximum extent of DMD—several statistical challenges were addressed. The outcome variable exhibited substantial zero inflation, and the limited sample size restricted the number of predictors that could be reliably incorporated into a conventional multivariable model; a two-part model was therefore employed. Variable selection for both components of the model was performed using least absolute shrinkage and selection operator (LASSO) regression, a method suitable for high-dimensional data and small sample sizes owing to its ability to minimise overfitting. In addition, sensitivity analyses were performed in which anterior chamber depth (ACD) was included in the models on the basis of clinical relevance. Given the constraints of the study, these analyses were considered exploratory and were intended to identify potential associations for future research.

Because both eyes from the same patient could be included, eye-level observations were not assumed to be independent. In both components of the two-part model, eyes were treated as observations clustered within patients, and a patient-specific random intercept was included using the patient identification number as the subject variable. The two-part model was specified as follows. Part 1 (DMD occurrence): a multivariable multilevel logistic regression model was used to estimate the odds of DMD occurrence (DMD extent > 0) versus no DMD. Part 2 (DMD extent): conditional on the presence of DMD, a second model analysed factors influencing its severity; this analysis was restricted to eyes with non-zero DMD values. The DMD extent was natural-log-transformed [ln(DMD extent)] to better approximate a normal distribution, and a multivariable multilevel linear regression model was applied to the transformed values.

All statistical analyses were performed using R version 4.4.1 (R Foundation for Statistical Computing, Vienna, Austria). A two-sided *P* value of less than 0.05 was considered statistically significant.

## Results

### Characteristics of the patients

A total of 50 patients (100 eyes) were included in the study cohort, comprising 18 (36%) males and 32 (64%) females, with a mean age of 26.68 ± 4.92 (21–38) years. The mean spherical power was −7.50 (−16.00, −1.50) D, the mean cylindrical power was −0.50 (−2.00, 0) D, and the mean spherical equivalent (SE) was −7.88 (−16.25, −1.50) D. The preoperative mean uncorrected distance visual acuity (UDVA) and corrected distance visual acuity (CDVA) were 1.4 (0.7, 1.7) logMAR and 0 (−0.2, 0.7) logMAR, respectively. Other ocular parameters included a mean endothelial cell density (ECD) of 3,048 (2,461, 3,933) cells/mm^2^, intraocular pressure (IOP) of 15 (10, 14) mmHg, and anterior chamber depth (ACD) of 3.17 (2.80, 3.72) mm. The demographic data and preoperative assessments are presented in Table 1.

**Table 1:** Baseline characteristics of the study patient.

Characteristics	Descriptive statistics: Oculus Dexter All(*n* = 100)	
Age (years), median (range)	26.68 ± 4.92 (21, 38)	
Sex, *n* (%)		
Male	18 (36.00%)	
Female	32 (64.00%)	
**Eye-level**	**OD**	**OS**
Preoperative Spherical Power, median (range)	−7.75(−16.00, −2.50)	−7.25(−15.50, −1.5)
Preoperative Cylindrical Power, median (range)	1.00 (0.10, 1.90)	1.10 (0, 2.10)
Preoperative diopter SE(D), median (range)	−8.13(−16.25, −3.00)	−7.63(−15.75, −1.5)
Preoperative UDCA(LogMar), median (range)	1.4 (0.7, 1.7)	1.4 (0.7, 1.7)
Preoperative CDCA(LogMar), median (range)	0(−0.2, 0.7)	0(−0.2, 0.3)
Preoperative IOP (mmHg), median (range)	14 (10, 14)	15 (11, 14)
Preoperative ACD (mm), median (range)	3.17 (2.8, 3.72)	3.17 (2.81, 3.71)
Preoperative WTW (mm), median (range)	11.65 (10.80, 12.90)	11.60 (10.60, 12.80)
Preoperative CCT (mm), median (range)	528.00 (454.00, 597.00)	524.50 (452.00, 601.00)
Preoperative axial length (mm), median (range)	26.66 (23.64, 30.03)	26.52 (23.23, 29.88)
Preoperative corneal ECD, median (range)	3,079.50 (2,546.00, 3,933.00)	3,030.00 (2,461.00, 3,764.00)
ICL degree SE(D), median (range)	−9.00(−17.00, −3.50)	−8.75(−16.50, −2.25)

### UDVA and efficacy index

At 1 month postoperatively, 98% of eyes achieved a UDVA equal to or better than the preoperative CDVA (Table 2). The postoperative UDVA at 1 month was −0.1 (−0.2, 0.5) logMAR, which was significantly better than the preoperative UDVA of 1.4 (0.7, 1.7) logMAR (*P* < 0.001).

**Table 2:** Comparison of preoperative and postoperative refractive parameters.

Outcomes	Before surgery	1 month after surgery	*P*
Sphere, D			
Overall	−7.50(−16.00, −1.50)	0.25(−1.00, 1.25)	<0.001
OD	−7.75(−16.00, −2.50)	0.50(−1.00, 1.25)	<0.001
OS	−7.25(−15.50, −1.50)	0.25(−1.00, 1.25)	<0.001
Cylinder, D			
Overall	−0.50(−2.00, 0)	−0.25(−2.00, 0)	<0.001
OD	−0.50(−2.00, 0)	−0.25(−2.00, 0)	<0.001
OS	−0.50(−1.75, 0)	−0.25(−1.50, 0)	<0.001
SE, D			
Overall	−7.88(−16.25, −1.50)	0.25(−1.25, 1.00)	<0.001
OD	−8.13(−16.25, −3.00)	0.25(−1.25, 1.00)	<0.001
OS	−7.63(−15.72, −1.50)	0.25(−1.25, 1.00)	<0.001
UCDA			
Overall	1.4 (0.7, 1.7)	−0.1(−0.2, 0.5)	<0.001
OD	1.4 (0.7, 1.7)	−0.1(−0.2, 0.5)	<0.001
OS	1.4 (0.7, 1.7)	−0.1(−0.2, 0)	<0.001

### Intraoperative distribution of DMD

The cumulative number of eyes in which DMD was detected by the end of each surgical step is shown in Supplementary Figure S1. No DMD was detected at the end of incision creation. By the end of ICL implantation, DMD had been detected in 21 eyes (21%), involving the anterior lip in 18 eyes and the posterior lip in 3 eyes. By the end of ICL positioning, the cumulative number increased to 34 eyes (34%), involving the anterior lip in 2 eyes and the posterior lip in 32 eyes. By the end of viscoelastic agent removal, DMD had been detected in a cumulative total of 45 eyes (45%), involving the anterior lip in 15 eyes and the posterior lip in 30 eyes. The cumulative number of eyes with DMD at each surgical step is also presented in Supplementary Figure S2.

DMD was most likely to occur during ICL implantation, probably due to friction between the delivery device and the anterior lip of the incision, and during ICL positioning, potentially due to contact between the positioning hook and the posterior lip. Only two eyes developed DMD involving both the anterior and posterior lips simultaneously, both during viscoelastic agent removal (Supplementary Figure S3); this may be related to irritation of the tunnel incision by the irrigation needle and IOP fluctuations caused by fluid exchange in the anterior chamber.

## Discussion

In the present study, the overall incidence of DMD at the surgical incision site during ICL surgery was 63%, which is lower than the 96% incidence of DMD observed in cataract surgery at Zhongshan Ophthalmic Center (18). This discrepancy may be attributable to the relatively small number of surgical steps required in phakic intraocular lens implantation and the absence of ultrasonic energy, which together substantially reduce the incidence of DMD.

The incidence of DMD in the present series should be interpreted in light of the wide range reported in the literature. In cataract surgery, clinically detected DMD occurs in 0.044%–0.5% of eyes after phacoemulsification and in up to approximately 2.5% after extracapsular cataract extraction, as summarized in previous comprehensive reviews (38). The higher incidence observed in ICL series, including the present study and the report from China, may be explained in part by the use of real-time intraoperative OCT, which detects small, clinically silent detachments that would be missed on routine slit-lamp examination, as well as by differences in incision construction and ICL implantation mechanics.

We analysed baseline patient characteristics, including age, IOP, axial length, cylindrical power, central corneal thickness, and surgical incision location. A statistically significant correlation was observed between IOP and the occurrence of DMD (*p* < 0.05) (Table 3, Part 1). However, because intraoperative IOP was not monitored during the study, no evidence could be obtained regarding the relationship between DMD and intraoperative IOP. Based on the occurrence of DMD (Table 3, Part 2), we also analysed whether age, cylindrical power, axial length, and anterior chamber depth (ACD) were correlated with DMD. Cylindrical power showed a possible association with DMD, although it did not reach statistical significance (*p* = 0.16), which may be attributable to the limited number of patients, the statistical methods employed, or other factors. Further studies enrolling larger cohorts are warranted to clarify the potential association between corneal astigmatism and DMD and to provide insights for future investigations.

**Table 3:** Multivariable two-part model of predictors for the occurrence and extent of DMD.

Predictors^a^	OR/β (95% confidence interval)	*P*
Part 1: Occurrence (binary variable)	1.004 (0.990, 1.019)	0.58
Anterior Chamber Depth	0.452 (0.076, 2.678)	0.37
Part 2: Extent (log-transformed continuous variable)		
Age	0.030(−0.016, 0.077)	0.19
Cylindrical power	0.358(−0.107, 0.824)	0.12
Axial Length	−0.046(−0.229, 0.137)	0.60
Anterior Chamber Depth	−0.453(−1.419, 0.513)	0.33

DMD, Descemet membrane detachment; OR, odds ratio.

a variable selection for both parts of the model was performed using the Least Absolute Shrinkage and Selection Operator regression method. Both components of the two-part model included a patient-specific random intercept, with the patient identification number specified as the clustering variable. The random-effects covariance matrix was specified as unstructured (TYPE = UN) to account for the correlation between fellow eyes.

### Real-time intraoperative OCT detection of DMD

Factors that can influence DMD in intraocular surgery generally include instrument-related, incision-related, and surgeon-related factors (25–27). In other intraocular procedures, such as vitreoretinal surgery, corneal transplantation, and cataract surgery (28–32), incision-site DMD may occur as a result of instrument sharpness, relatively older patients, poor ocular tissue conditions, and poor patient cooperation. Conventional microscopes can only visualize the corneal surface, making it difficult to identify tiny, peripheral, or localized DMD; once mild corneal edema develops, structural discrimination becomes even more challenging. The intraoperative OCT navigation microscope can directly display the separation between the Descemet membrane and the corneal stroma. Even small or peripheral detachments can be detected promptly, thereby avoiding missed diagnosis of subclinical detachment and enabling early intraoperative identification of complications.

No DMD was detected by the end of incision creation. The cumulative number of eyes with DMD increased to 21 (21%) by the end of ICL insertion, 34 (34%) by the end of ICL positioning, and 45 (45%) by the end of irrigation/aspiration of the viscoelastic agent. Throughout the procedure, intraoperative OCT enabled the surgeon to detect DMD more promptly.

Previous animal studies have shown that the continuous vibration and mechanical activity of the phacoemulsification needle can cause detachment of the Descemet membrane at the incision site during phacoemulsification (33). In recent years, case reports on DMD (34) and studies of femtosecond laser-assisted cataract surgery (35) have reported a DMD incidence of 3.9%, with risk factors including an endothelial cell density below 2,000 cells/mm^2^ and corneal granular lesions. In the present study, a relatively large number of patients developed DMD during thorough removal of the viscoelastic agent. The sustained occurrence of DMD during this step was attributed to the viscoelastic agent, a high-molecular-weight sodium hyaluronate, which mostly flowed with the irrigation toward the surgical incision and was washed out of the anterior chamber during rinsing. In a few patients, the viscoelastic agent was trapped near the peripheral anterior chamber angle between the cornea and iris, requiring repeated changes in the direction and angle of the irrigation needle to create a swirling flow and wash it out; some of these patients required a relatively long rinsing time. We speculate that repeated changes in the direction and angle of the irrigation needle may have impacted the endothelium of the anterior or posterior corneal lip, leading to the occurrence of DMD.

To further clarify whether DMD occurs during ICL surgery and which surgical step has the greatest impact on it, we introduced intraoperative OCT navigation microscopy. This represents the latest generation of surgical microscopes, combining the LUEMRA microscope platform with OCT technology to enable real-time observation of OCT images during surgery. Previous studies have reported the use of intraoperative OCT navigation microscopy to observe the vaulting condition and intraoperative changes during vitreoretinal surgery, corneal transplantation, cataract surgery, and phakic intraocular lens implantation (36). Recent studies have also used this technique to observe the condition at the incision site during cataract surgery (39). To our knowledge, the present case series is the first study to use intraoperative OCT navigation to dynamically assess changes in DMD at the 3.0 mm surgical incision site during phakic intraocular lens implantation.

The ACD of these patients was 3.17 (3.00–3.33) mm. No statistically significant correlation was found between ACD and DMD, suggesting that DMD may be related not to ACD but rather to the structure of the incision, the size of the internal opening, or the injection angle, which may cause friction against the anterior corneal lip.

### Clinical implications and educational value

In the present study, although the overall proportion of DMD was lower than that in cataract surgery (40), some patients still developed DMD during ICL implantation and viscoelastic removal, owing to the smaller operating space in phakic intraocular lens implantation compared with cataract surgery. Although DMD after phakic intraocular lens implantation rarely leads to poor vision, we still recommend that novice surgeons pay close attention to the direction and angle of instruments during surgery to avoid DMD.

Based on the present findings, several strategies may help reduce intraoperative DMD. For example, if significant resistance is encountered while creating the incision, the surgeon should immediately abandon the current tunnel knife and use a sharper blade. The configuration of the surgical incision can be optimized to prevent DMD caused by mechanical friction. The incision size should match the injector size as closely as possible, because an excessively small incision increases friction between the injector and the incision and may lead to DMD. Among the four surgical steps, viscoelastic removal was associated with the largest number of patients developing DMD and the largest number with prolonged DMD. We therefore recommend that surgeons minimize the volume of viscoelastic agent injected to reduce the occurrence and progression of DMD during its removal. For patients with lower preoperative IOP, particular attention should be paid to maintaining a relatively high intraoperative IOP, which may reduce the occurrence of DMD.

### Limitations and future perspectives

The principal strength of this study is the sequential, step-by-step assessment of DMD during ICL surgery without phacoemulsification, which enables real-time characterization of DMD development and progression throughout the procedure. Given the current scarcity of data on how individual intraoperative steps influence DMD, this real-time assessment has direct implications for clinical practice and ICL surgical safety.

Nevertheless, several limitations should be acknowledged. First, all procedures were performed by a single experienced, high-volume ICL surgeon. Although this ensures technical standardization, it may also have contributed to a relatively low DMD incidence and restricts the extrapolation of our findings to less experienced or low-volume surgeons. A recent study comparing phacoemulsification performed by residents versus professional staff reported a higher incidence of incision-site DMD in the resident group (41), supporting surgeon experience as an important determinant of this complication.

Second, stage-related DMD outcomes were summarized cumulatively, because DMD detected at an earlier stage may persist or extend during subsequent manipulation. Although this approach appropriately captures the progressive proportion of eyes affected during surgery, it does not permit estimation of the independent incidence or causal contribution of each stage; formal comparisons of stage-specific risks were therefore beyond the scope of this study.

Third, all DMD measurements were obtained using ImageJ software and evaluated by a single observer. The results may thus be susceptible to observer-related variability or bias and should be interpreted and generalized with caution, as they are partly dependent on the professional skills and judgment of one observer.

Fourth, patients with poor corneal or ocular surface conditions were excluded. Previous studies have indicated that ocular surface injury and associated changes involving the corneal limbus and Bowman layer may contribute to DMD (37, 38): histologically, inflammatory cell infiltration in the Bowman layer and fluid exudation involving the Descemet membrane have been described, which may alter corneal morphology and endothelial function and thereby promote DMD. Consequently, the present findings may not be generalizable to patients with compromised corneal conditions, and future studies should evaluate surgical outcomes in this population.

Fifth, this was a single-center study with a relatively small number of eyes, which further limits the generalizability and precision of the estimates. Finally, the follow-up duration and the number of postoperative assessment time points were limited.

Future multicenter prospective studies incorporating larger samples, surgeons of varying experience levels, standardized assessment by multiple masked observers, prespecified stage-specific outcomes, patients with compromised corneal conditions, and longer follow-up are warranted to confirm and extend the present findings.

## Conclusions

Intraoperative OCT navigation microscopy enables real-time visualization of the surgical incision during ICL surgery. Our observations demonstrated that the angle at which surgical instruments engaged the incision was most strongly associated with DMD occurrence, and that DMD was detected most frequently during viscoelastic agent removal. These findings suggest that the volume of viscoelastic agent injected should be minimized, thereby reducing the risk of DMD occurrence and progression during its subsequent removal.

## Data Availability

The original contributions presented in the study are included in the article/Supplementary Material, further inquiries can be directed to the corresponding authors.

## Supplementary material

Supplementary Figure S1 Distribution of intraoperative Descemet membrane detachment by surgical phase and location.

Supplementary Figure S2 Distribution of Intraoperative Descemet Membrane Detachment by Surgical Phase (n = 100).

Supplementary Figure S3 Distribution of intraoperative Descemet membrane detachment by location (n = 100).
